# Influences of Playfulness on Smartphone Dependency among Upper Grades of Korean Elementary Schoolers

**DOI:** 10.3390/ijerph19127185

**Published:** 2022-06-11

**Authors:** Seong Eon Kim, Hyoung-Kil Kang

**Affiliations:** 1 Department of Police Science, Kyungnam University, Changwon 51767, Korea; seonkim@kyungnam.ac.kr; 2 Department of Physical Education, Kyungnam University, Changwon 51767, Korea

**Keywords:** social engagement playfulness, physical animation playfulness, emotional fluidity playfulness, smartphone dependency, elementary schoolers

## Abstract

A paucity of research has addressed the relationship between each psychological construct of playfulness and smartphone dependency, and the purpose of this research is to understand how each psychological construct of playfulness, including physical animation, social engagement, mental spontaneity, emotional fluidity, and humorous perspective playfulness, influences smartphone dependency of the upper grades of elementary schoolers. For this purpose, a total of 278 questionnaires was analyzed for descriptive, correlation, and multiple regression analysis. The correlation analysis showed that respondents’ age positively and parents’ education levels negatively correlates to smartphone dependency. The multiple regression analyses showed that physical animation playfulness and emotional fluidity playfulness negatively and social engagement playfulness positively influence smartphone dependency of the respondents. The findings indicate that to reduce smartphone dependency among elementary schoolers, physical animation and emotional fluidity playfulness need to be promoted. The findings also suggest that each component of playfulness has distinctive advantages and disadvantages of developmental processes in childhood, and more future research endeavors need to be directed to understand the role of playfulness in children’s behaviors and cognitive processes.

## 1. Introduction

In 2020, about 95% of adolescents and 93% of 6th graders in South Korea owned a smartphone, which is the highest rate in the world, and 30.5% of them experienced smartphone dependency [1]. Although some researchers refer to smartphone addiction, this term has a focus on pathology and overly ignores the benefits of smartphone use. “Smartphone dependency” avoids these difficulties and is the term adopted in the present study. In research fields, the excessive use of a smartphone has been referred to as smartphone addiction, but this study adopts the term smartphone dependency because smartphone addiction presumes that all smartphone users are potentially pathological addicts, overly ignoring the benefits of smartphone use [2]. Adolescents with smartphone dependency feel uneasy and anxious when a smartphone is not readily available, and the rate of smartphone dependency of Korean adolescents was relatively stable from 2016 to 2019, ranging from 29.3% to 30.6% [1]. However, during the COVID-19 period from 2019 to 2020, it sharply increased by 5.2% from 30.6% to 35.8% and has become a timely issue [1].

Previous studies have sought to investigate the causes of the smartphone dependency among adolescents and have found that self-efficacy, self-control, and school life satisfaction decrease smartphone dependency, while aggression, social withdrawal, and academic helplessness contribute to increasing smartphone dependency among adolescents [3,4]. However, there is a relative paucity of research addressing the smartphone dependency of the upper grades of elementary schoolers who are in the fourth to sixth grades although they are vulnerable to smartphone dependency.

The upper grades of elementary schoolers experience significant developmental changes in the cognitive, social, and affective domains, and they are vulnerable to be exposed to smartphone dependency, as they have immature behavioral control skills and become more interested in social and cultural contents [5,6]. Studies showed that their smartphone dependency is negatively associated with peer relationships and communication with parents and positively correlated with carpal tunnel syndrome, turtleneck syndrome, depression, and anxiety [7]. More importantly, elementary schoolers’ smartphone dependency increases the likelihood to be pathologically addicted to smartphones in their adulthood [8]. With the concerns of the prevalence and lifelong negative effects of the smartphone dependency among the upper grades of elementary schoolers, this study is the first to focus on the influences of playfulness on smartphone dependency among upper-grade Korean elementary students. The next section explains the conceptual framework for the present study.

### 1.1. Playfulness

Play is defined as “voluntary activity pursued without ulterior purpose and, on the whole, with enjoyment or expectation of enjoyment” [9] (p. 394). To perceive an activity as play, people must have the desire to play and must internally perceive an activity as play [10,11]. This disposition is playfulness, which is the essence of play [12], and the definition of playfulness is “… an individual differences variable that allows people to frame or reframe everyday situations in a way such that they experience them as entertaining, and/or intellectually stimulating, and/or personally interesting” [13] (p. 114). That is, play is a behavior and activity, but playfulness is a type of personality and an individual’s specific predisposition to engage in play behavior [11]. People with high playfulness enjoy interacting playfully with others and using their playfulness to resolve tension in difficult situations, and playfulness is also positively related to a preference for unique and unusual activities and a preference for complexity rather than simplicity [13]. A lack of playfulness in a child may reflect cognitive, emotional, and physical deficits, and creativity, spontaneity, and social connection to others are positively associated with playfulness [14]. Lieberman [11] asserted that a playful approach to life can be beneficial for people of all ages because people can enjoy, learn, and obtain perspective in a comfortable manner that might be only possible with such a playful attitude. As such, playfulness provides information regarding individuals’ behavior and cognitive style and can be considered a source of individual differences, similar to personality [15].

With this notion, Staempfli [16] proposed five psychological components of playfulness, which influence individuals’ behaviors and cognitive styles. The five psychological components of playfulness are associated with mental spontaneity, social engagement, physical animation, humorous perspective, and emotional fluidity. Kim and colleagues [17] summarized the five components. Mental spontaneity is relevant to creativity and imagination, and people with strong mental spontaneity like to imagine themselves in funny situation, enjoy playing role games, and tend to attempt unexperienced games. People with high social engagement enjoy socializing with others, and their preference for play selection is closely associated with the levels of the interaction between players. People with strong physical animation want to be physically active and are motivated by being physically active. People with strong humorous perspective find it easy to find something to laugh at in difficult situations and like to hear and tell funny stories. Lastly, people with emotional fluidity smile and laugh regularly and more often and freely express their pleasure and emotions. These five psychological components of playfulness can differently influence people’s decisions and behaviors [16,17]. However, to the best of our knowledge, there is no study addressing how each psychological component of playfulness influences smartphone dependency among the upper grades of elementary schoolers although the effects of playfulness on people’s decisions and behaviors become stronger in informal, familiar, or private situations, where most of the use of smartphones occurs [18]. Thus, this study aims to examine the relationships between the five psychological constructs of playfulness and smartphone dependency and smartphone dependency, consisting of four components.

### 1.2. Smartphone Dependency

Smartphone dependency consists of four psychological components, and the four components are disturbance of adaptive functions, virtual life orientation, withdrawal, and tolerance [19]. The disturbance of adaptive functions relates to daily life disorders due to the excessive use of smartphone, such as having a hard time doing homework and executing a study plan. People with strong virtual life orientation tend to think that smartphone use is more enjoyable than time spent with family or friends and feel lost in the real world if they are not able to access their smartphone. People with the strong withdrawal feel restless and anxious and panic when they do not have their smartphone. Lastly people with tolerance issue cannot control their smartphone usage time even when they think they should stop using it. These four components of smartphone dependency can be differently affected by the five components of playfulness, and the purpose of this study is to examine the relationships between psychological constructs of playfulness and smartphone dependency. For the purpose, the following research question is presented: How does each of the five components of playfulness (physical animation, social engagement, mental spontaneity, emotional fluidity, and humorous perspective) influence the four components of smartphone dependency (disturbance of adaptive functions, virtual life orientation, withdrawal, and tolerance)?

## 2. Materials and Methods

### 2.1. Participants

This study used a cross-sectional design and a convenience sampling approach to recruit subjects. In 2019, questionnaires were distributed by teachers to a total of 300 students in five elementary schools in Incheon city, and the written consents of students and their parents were obtained prior to their participation in this study. After the exclusion of 22 incomplete questionnaires, 278 completed questionnaires were coded and used for data analysis. The students participating in this study reported their age, height, weight, gender, father’s education, and mother’s education, and based on self-reported height and weight, participants’ body mass index (BMI) was calculated. The average age and BMI of the participants was 11.93 years old and 18.32, respectively, and the gender distribution of the study participants was 126 male students (45.65%) and 150 female students (54.34%). Eighty-one percent of the participants’ fathers and 76% of the participants’ mothers completed a Bachelor’s degree. The summary of the characteristics of the participants is presented in Table 1.

### 2.2. Measures

In addition to five items measuring age, height, weight, father education, and mother’s education, items developed by Staempfli [16] were used to measure playfulness. The five components of playfulness consist of 20 items, and the playfulness items used a 5-point Likert scale: (1) strongly disagree; (2) disagree; (3) neither agree nor disagree; (4) agree; and (5) strongly agree. In detail, the physical animation playfulness items consist of the following: “I like to play and horse around with my friends”; “When I hang out with friends, we usually like to play around”; “I like to be active physically”; and “Being physically active keeps me stimulated and motivated”. The social engagement playfulness items include “By being playful, it is easier to get along with people”; “I like to interact with people in a playful way”; “I like to make people laugh”; and “I feel comfortable joking around with others”. The mental spontaneity playfulness items consist of the following: “I like to imagine myself and other people in funny situations”; “I like to play with ideas”; “I have an active imagination”; and “I like to imagine myself as being different people or different characters”. The emotional fluidity playfulness items include “I like to sing and hum out loud when I am happy”; “I laugh and smile a lot”; “My friends can tell when I am having a good time”; and “In most situations, I express my emotions freely”. The humorous perspective items include “I like to clown around”; “I can usually find something to laugh and joke about in difficult situations”; “I can find something comical or humorous in most situations”; and “I like to tell funny stories”.

To measure smartphone dependency, 15 items developed by Kim and his colleagues [17] were used, and the 15 smartphone dependency items employed a 4-point Likert scale: (1) strongly disagree; (2) disagree; (3) agree; and (4) strongly agree. The five items for measuring disturbance of adaptive functions are “My school grades dropped due to excessive smartphone use”; “I have a hard time doing what I have planned (study, do homework, or go to afterschool classes) due to using smartphone”; “People frequently comment on my excessive smartphone use”; “Family or friends complain that I use my smartphone too much”; and “My smartphone does not distract me from my studies (reverse-coded item)”. The two items for measuring virtual life orientation are “Using a smartphone is more enjoyable than spending time with family or friends” and “When I cannot use a smartphone, I feel like I have lost the entire world”. The four items for measuring withdrawal are “It would be painful if I am not allowed to use a smartphone”; “I get restless and nervous when I am without a smartphone”; “I am not anxious even when I am without a smartphone (reverse-coded item)”; and “I panic when I cannot use my smartphone”. To measure tolerance, four items were used, including “I try cutting my smartphone usage time, but I fail”; “I can control my smartphone usage time (reverse-coded item)”; “Even when I think I should stop, I continue to use my smartphone too much”; and “Spending a lot of time on my smartphone has become a habit”.

## 3. Results

### 3.1. Correlation Analysis

A correlation analysis was conducted on the study variables as presented in Table 2.

Age is significantly positively correlated with the three components of smartphone dependency, including virtual life orientation (*r* = 0.139, *p* < 0.05), withdrawal (*r* = 0.194, *p* < 0.01), and tolerance (*r* = 0.124, *p* < 0.05). Father’s education is significantly positively correlated with mental spontaneity of playfulness (*r* = 0.126, *p* < 0.05). Mother’s education is significantly positively correlated with all the components of playfulness, including physical animation playfulness (*r* = 0.278, *p* < 0.01), social engagement playfulness (*r* = 0.225, *p* < 0.01), mental spontaneity playfulness (*r* = 0.172, *p* < 0.01), emotional fluidity playfulness (*r* = 0.197, *p* < 0.01), and humorous perspective playfulness (*r* = 0.160, *p* < 0.05), and is significantly negatively correlated with disturbance of adaptive functions (*r* = −0.131, *p* < 0.05) and withdrawal (*r* = −0.128, *p* < 0.05) of smartphone dependency. All the components of playfulness are significantly positively correlated with each other, but multicollinearity issues between the variables were not identified, as every correlation between the variables is lower than 0.80 [18]. Physical animation of playfulness is significantly negatively correlated with virtual life orientation (*r* = −0.132, *p* < 0.05) of smartphone dependency, and emotional fluidity of playfulness is significantly negatively correlated with disturbance of adaptive functions (*r* = −0.123, *p* < 0.05), virtual life orientation (*r* = −0.171, *p* < 0.01), and withdrawal (*r* = −0.166, *p* < 0.01) of smartphone dependency. All the components of smartphone dependency are positively correlated with each other.

### 3.2. Multiple Regression Analyses

Four multiple regression analyses were conducted, and the results of the four multiple regression analyses are summarized in Table 3. In the multiple regression analyses, all the five components of playfulness were served as independents variables, and each of the four components of smartphone dependency was served as a dependent variable. The multiple tolerance values and VIF values were accessed to find multicollinearity issues, and multicollinearity issues were not identified in all the four multiple regression analyses [20,21].

The multiple regression analyses showed that physical animation playfulness (*β* = −0.185, *t* = −2.283, *p* < 0.05) and emotional fluidity playfulness (*β* = −0.243, *t* = −2.834, *p* < 0.01) significantly negatively influence virtual life orientation smartphone dependency, and social engagement playfulness (*β* = 0.225, *t* = 2.184, *p* < 0.05) significantly positively influences virtual life orientation smartphone dependency. Emotional fluidity playfulness (*β* = −0.264, *t* = −3.080, *p* < 0.01) significantly negatively influences withdrawal smartphone dependency, and physical animation playfulness (*β* = −0.198, *t* = −2.430, *p* < 0.05) significantly negatively influences tolerance smartphone dependency. Lastly, social engagement playfulness (*β* = 0.296, *t* = 2.833, *p* < 0.01) significantly positively influences tolerance smartphone dependency.

## 4. Discussion

This study examined the influence of playfulness on smartphone dependency among the upper grades of Korean elementary schoolers. The correlation analyses show that as elementary schoolers move from fourth to sixth grades, their smartphone dependency increases, and as parents’ education levels increases, their smartphone dependency decreases. This finding is consistent with the recent report of Korean government national study presenting that the rate of smartphone dependency increases from elementary schoolers to middle school students, and it decreases as household income, which is positively associated with parents’ education levels, increases [1]. The correlation analysis also shows that as parents’ education levels increase, their children’s playfulness increases. This finding can be inferred in that adults’ educational success is found to be positively related to their playfulness, and parents’ playfulness is positively connected to their children’s playfulness [22,23]. It is interesting that mother’s education levels are correlated to all subfactors of playfulness, while father’s education levels are only correlated with mental spontaneity playfulness. This finding can be not only that, in Korea, mothers simply spend more times with their children than fathers but also that mothers and fathers differ in the quality of play activity with their children. It is reported that when mothers play with their children, they encourage their children’s negotiation skills and shared decision making and tend to allow their children to modify play activities and to use objects in creative ways to make play activities more fun [24]. These differences of the quality of play activity between mothers and fathers as well as the different lengths of time with their children can contribute to differentiating mothers’ and fathers’ influences on children’s playfulness.

To the best of our knowledge, there is no study addressing the direct relationships between each of the psychological constructs of playfulness and smartphone dependency among elementary schoolers. Thus, the findings of multiple regression analyses of this study are of an initial and exploratory nature and can be discussed presumably with similar concepts. With this notion, the following discussion is described. This study found that respondents with high emotional fluidity show low virtual life orientation smartphone dependency and withdrawal smartphone dependency, and emotional expressiveness and depression can be employed to discuss the finding. The items of emotional fluidity playfulness are “I laugh and smile a lot”; “I like to sing and hum out loud when I am happy”; “My friends can tell when I am having a good time”; and “In most situations, I express my emotions freely”. All the four items are closely relevant to emotional expressiveness and more frequently occur when people are not depressed. Studies identified that emotional expressiveness is an antecedent of addictive behaviors because people with difficulties in expressing their emotions are immature to deal with and more suffer more from their negative emotions and have high tendency to become dependent on their smartphone, the Internet, and alcohol [25]. It was reported that inability to express emotions is significantly higher in the alcohol-dependent group than in the non-dependent group, and emotional expressiveness negatively correlates with Internet addiction [25,26]. It was also found that immature emotional expressiveness is associated with low self-concept, which increases smartphone dependency, and people with difficulty in expressing emotions are more prone to be dependent on smartphones because they heavily rely on Social Networking Service (SNS) to maintain their social relationships [25,26]. With regard to depression and smartphone dependency, individuals with depression symptoms tend to overuse smartphones to reduce their psychological distress, and the longitudinal and bidirectional relationships between depressive symptoms and smartphone dependency are reported [27,28]. It is also found that life satisfaction and happiness positively relate to playfulness, and happiness is negatively associated with smartphone dependency [29]. Collectively, smartphone dependency is negatively related to emotional expressiveness and positively connected to depression, and in this sense, the current study finding of the negative association between emotional fluidity playfulness and smartphone dependency can be interpreted.

The multiple regression analyses also showed that physical animation playfulness significantly negatively relates to the virtual life orientation smartphone dependency and tolerance smartphone dependency among the upper grades of Korean elementary schoolers. The physical animation playfulness items are “I like to play and horse around with my friends’; “When I hang out with friends, we usually like to play around”; “I like to be active physically”; and “Being physically active keeps me stimulated and motivated”. As in the description of the items, the physical animation playfulness is related to preference for physically active plays and can be positively associated with how the respondents are physically active in their life. The use of smartphones is known to promote sedentary behaviors because smartphone functions such as texting messages, updating SNS, browsing the Internet, and playing mobile games have been historically considered sedentary behaviors that promote smartphone dependency [30]. That is, the lower the respondents score in physical animation playfulness, the greater physically inactivity they display, and the higher their chances to experience smartphone dependency. It is also reported that the use of smartphones interrupts physical activity and especially reduces cardiorespiratory fitness such as peak oxygen consumption, and people with high-risk smartphone dependency have a significantly lower number of walking steps and muscle mass and have a higher number of fat mass than those with no risk and potential risk of smartphone dependency [31]. These previous studies indicate that smartphone dependency positively relates to obesity among children, and obesity negatively correlates to playfulness [31]. Furthermore, physically inactive individuals experience higher levels of perceived stress, and physical activity induces psychological benefits such as resilience and self-esteem, which help to cope with stress, which is one of the main causes of smartphone dependency [32,33]. For all these reasons, physical animation playfulness physiologically and psychologically reduces the likelihood of smartphone dependency among the upper grades of elementary schoolers, and from this perspective, the current study finding can be understood.

This study found that social engagement playfulness increased virtual life orientation smartphone dependency and withdrawal smartphone dependency. The items of the social engagement playfulness are “By being playful it is easier to get along with people”; “I like to interact with people in a playful way”; “I like to make people laugh”; and “I feel comfortable joking around with others”. In the consideration of the description of the items, it is inferred that respondents with high social engagement playfulness highly value social behavior and interpersonal relationships, and the current finding of the relationships can be understood with friendships in online and offline spaces and social smartphone use. Yau and Reich [34] reported that adolescents’ digital interaction behaviors are similar to their offline ones, and adolescents use technology-mediated communication platforms to extend and complement their offline interactions rather than replace them. The social engagement playfulness items in this study are designed to measure respondents’ preferences for offline social behaviors, and their preferences seem to remain the same in the online social interactions, which potentially links to the excessive use of smartphones. In addition, smartphone social interactions provide benefits over offline interactions in developing friendships. For example, sharing intimate information via smartphone is more preferred than in-person because text messages provides users with more time to craft their responses and to control their emotions [35]. Smartphones are also effective in promoting companionship between offline friends because smartphones allow constant communication and interaction by circumventing the common restrictions of offline communication, such as a loud volume in a concert and adult monitoring [36]. As such, respondents with high social engagement playfulness may use smartphone as a means of cultivating their friendships and are more likely to indulge themselves in the excessive use of smartphones. It is also expected that respondents with high social engagement playfulness more frequently use Facebook and LINE, i.e., social smartphone use. Previous studies reported a positive relationship between social smartphone use and smartphone dependency because in social smartphone use, people can make new friends without in-person communication, which can decrease social anxiety, enhance social comfort, and easily increase their number of friends, and this notion supports the finding of the current study [37,38,39,40].

The current study is with its limitations. First, self-administered questionnaires are susceptible to be biased, such as recall biases and social desirability, and future studies need to consider multi-method assessments to measure elementary schoolers’ playfulness and smartphone dependency, including peers’ or teachers’ evaluation of playfulness types, parents’ observation-based smartphone dependency, and the mixed research design. Second, the respondents were sampled based on convenience sampling method, and caution needs to be taken to interpret the findings of the current study. Third, the present study was conducted cross-sectionally, and the interpretation of the directional relationships is not obvious. Future studies may use longitudinal research design for the better understanding of the causal relationships. Despite the limitations, for the best of the authors’ knowledge, this is the first study to investigate the relationships between each trait of playfulness and smartphone dependency among elementary schoolers, and this study thus can add innovative contribution to the literature.

## 5. Conclusions

Smartphone dependency has been sharply increased during the COVID-19 era and has become a timely issue in Korea. To the best of our knowledge, this study is the first study with the purpose of determining how the psychological constructs of playfulness, including physical animation playfulness, social engagement playfulness, mental spontaneity playfulness, emotional fluidity playfulness, and humorous perspective playfulness, influence smartphone dependency among the upper grades of Korean elementary schoolers. This study is based on the notion that playfulness is a personality trait that influences elementary schoolers’ behaviors, and it identified that some components of playfulness significantly and differently affect elementary schoolers’ smartphone dependency. Physical animation and emotional fluidity playfulness weakened smartphone dependency, while social engagement playfulness contributed to smartphone dependency. Some may consider that elementary schoolers’ playfulness creates disruptive and harmful behaviors in classrooms and at home [11,14]. However, this study found that each component of playfulness has distinctive advantages and disadvantages in the developmental processes of childhood. More future research endeavors need to be directed to understand the role of each component of playfulness in children’s behaviors and cognitive processes.

## Figures and Tables

**Table 1:** Characteristics of Study Participants.

Characteristics	Frequency	*M*	*SD*
Age (*n* = 278)	11 years old (*n* = 103, 37.05%) 12 years old (*n* = 106, 38.12%) 13 years old (*n* = 69, 24.82%)	11.93	0.87
Height (*n* = 267)		146.10 cm	12.31
Weight (*n* = 258)		39.11 kg	8.97
BMI (*n* = 258)		18.32	
Gender (*n* = 276)	Male (*n* = 126, 45.65%) Female (*n* = 150, 54.34%)		
Father’s Education (*n* = 264)	Elementary (*n* = 0, 0.00%)		
	Middle School (*n* = 1, 0.37%)		
	High School (*n* = 49, 18.56%)		
	Bachelor’s (*n* = 197, 74.62 %)		
	Master’s (*n* = 15, 5.68%)		
	Ph.D. (*n* = 2, 0.75%)		
Mother’s Education (*n* = 262)	Elementary (*n* = 2, 0.76%)		
	Middle School (*n* = 2, 0.76%)		
	High School (*n* = 57, 21.75%)		
	Bachelor’s (*n* = 196, 74.80%)		
	Master’s (*n* = 5, 1.91%)		
	Ph.D. (*n* = 0, 0.00%)		

**Table 2:** Correlations of Study Variables.

Study Variables	(1)	(2)	(3)	(4)	(5)	(6)	(7)	(8)	(9)	(10)	(11)	(12)	(13)	(14)
(1) Age	1	0.379 **	0.279 **	−0.075	−0.078	−0.020	0.025	−0.053	−0.099	0.009	0.114	0.139 *	0.194 **	0.124 *
(2) Height	0.379 **	1	0.543 **	−0.074	0.008	−0.003	0.050	−0.025	0.045	0.102	0.076	0.074	0.108	0.083
(3) Weight	0.279 **	0.543 **	1	−0.141 *	−0.074	0.020	0.012	0.039	−0.055	0.093	0.119	0.047	0.069	0.061
(4) Father’s Education	−0.075	−0.074	−0.141 *	1	0.460 **	0.035	0.066	0.126*	−0.030	0.063	−0.024	−0.014	0.052	−0.034
(5) Mother’s Education	−0.078	0.008	−0.074	0.460 **	1	0.278 **	0.225 **	0.172 **	0.197 **	0.160 *	−0.131 *	−0.086	−0.128 *	−0.087
(6) Physical Animation	−0.020	−0.003	0.020	0.035	0.278 **	1	0.648 **	0.420 **	0.505 **	0.440 **	−0.044	−0.132 *	−0.095	−0.081
(7) Social Engagement	0.025	0.050	0.012	0.066	0.225 **	0.648 **	1	0.602 **	0.636 **	0.641 **	−0.008	−0.018	−0.025	0.075
(8) Mental Spontaneity	−0.053	−0.025	0.039	0.126 *	0.172 **	0.420 **	0.602 **	1	0.581 **	0.523 **	−0.106	−0.040	−0.023	−0.019
(9) Emotional Fluidity	−0.099	0.045	−0.055	−0.030	0.197 **	0.505 **	0.636 **	0.581 **	1	0.536 **	−0.123 *	−0.171 **	−0.166 **	−0.091
(10) Humorous Perspective	0.009	0.102	0.093	0.063	0.160 *	0.440 **	0.641 **	0.523 **	0.536 **	1	−0.005	−0.015	−0.014	0.031
(11) Disturbance of adaptive functions	0.114	0.076	0.119	−0.024	−0.0131 *	−0.044	−0.008	−0.106	−0.123 *	−0.005	1	0.360 **	0.490 **	0.650 **
(12) Virtual Life Orientation	0.139 *	0.074	0.047	−0.014	−0.086	−0.132 *	−0.018	−0.040	−0.171 **	−0.015	0.360 **	1	0.659 **	0.377 **
(13) Withdrawal	0.194 **	0.108	0.069	0.052	−0.128 *	−0.095	−0.025	−0.023	−0.166 **	−0.014	0.490 **	0.659 **	1	0.446 **
(14) Tolerance	0.124 *	0.083	0.061	−0.034	−0.087	−0.081	0.075	−0.019	−0.091	0.031	0.650 **	0.377 **	0.446 **	1

* *p* < 0.05, ** *p* < 0.01.

**Table 3:** Multiple Regression Analysis of Playfulness and Smartphone Dependency.

DV	IV	*β*	*t*	*MT*	*VIF*	*F*	*R* ^2^
Disturbance of Adaptive Functions (*M* = 1.85, *SD* = 0.62, *N* = 256)	Physical Animation (*M* = 3.62, *SD* = 0.95)	−0.041	−0.500	0.573	1.745	1.387	0.027
	Social Engagement (*M* = 3.70, *SD* = 0.87)	0.102	0.962	0.350	2.860		
	Mental Spontaneity (*M* = 3.59, *SD* = 0.81)	−0.101	−1.210	0.561	1.784		
	Emotional Fluidity (*M* = 3.77, *SD* = 0.84)	−0.148	−1.693	0.511	1.956		
	Humorous Perspective (*M* = 3.13, *SD* = 1.00)	0.091	1.072	0.542	1.845		
Virtual Life Orientation (*M* = 1.30, *SD* = 0.50, *N* = 256 )	Physical Animation (*M* = 3.62, *SD* = 0.95)	−0.185	−2.283 *	0.574	1.743	3.314 **	0.062
	Social Engagement (*M* = 3.70, *SD* = 0.87)	0.225	2.184 *	0.353	2.834		
	Mental Spontaneity (*M* = 3.59, *SD* = 0.81)	−0.002	−0.023	0.565	1.769		
	Emotional Fluidity (*M* = 3.77, *SD* = 0.84)	−0.243	−2.834 **	0.509	1.963		
	Humorous Perspective (*M* = 3.13, *SD* = 1.00)	0.057	0.684	0.550	1.819		
Withdrawal (*M* = 1.33, *SD* = 0.51, *N* = 256)	Physical Animation (*M* = 3.62, *SD* = 0.95)	−0.106	−1.298	0.571	1.752	2.60 *	0.049
	Social Engagement (*M* = 3.70, *SD* = 0.87)	0.159	1.531	0.350	2.860		
	Mental Spontaneity (*M* = 3.59, *SD* = 0.81)	0.037	0.447	0.561	1.783		
	Emotional Fluidity (*M* = 3.77, *SD* = 0.84)	−0.264	−3.080 **	0.514	1.945		
	Humorous Perspective (*M* = 3.13, *SD* = 1.00)	0.064	0.767	0.545	1.836		
Tolerance (*M* = 1.85, *SD* = 0.71, *N* = 256 )	Physical Animation (*M* = 3.62, *SD* = 0.95)	−0.198	−2.430 *	0.571	1.751	2.81 *	0.053
	Social Engagement (*M* = 3.70, *SD* = 0.87)	0.296	2.833 **	0.348	2.877		
	Mental Spontaneity (*M* = 3.59, *SD* = 0.81)	−0.051	−0.612	0.556	1.800		
	Emotional Fluidity (*M* = 3.77, *SD* = 0.84)	−0.165	−1.923	0.514	1.945		
	Humorous Perspective (*M* = 3.13, *SD* = 1.00)	0.045	0.539	0.541	1.847		

* *p* < 0.05, ** *p* < 0.01.

## Data Availability

Not applicable.
